# Multiscale Peritumoral Ultrasound Machine Learning Scores and Pathological Response to Neoadjuvant Therapy in Breast Cancer

**DOI:** 10.3390/diagnostics16213580

**Published:** 2026-10-29

**Authors:** Ying Wang, Ameng Shi, Jun Lian, Runa Liang, Juan Wang, Jue Jiang

**Affiliations:** 1 Department of Ultrasound, Second Affiliated Hospital of Xi’an Jiaotong University, No. 157 Xiwu Road, Xi’an 710004, China; wangying2014@xjtu.edu.cn (Y.W.); shiameng13@163.com (A.S.); 2 Department of Ultrasound, Ankang City Central Hospital, No. 85 Jinzhou South Road, Ankang 725000, China; 18717578077@163.com (J.L.); lrn243483802@163.com (R.L.)

**Keywords:** breast cancer, peritumoral ultrasound, machine learning, neoadjuvant therapy, pathological response

## Abstract

**Background/Objectives:** Peritumoral ultrasound may capture tissue characteristics relevant to treatment sensitivity. In this study, we examined whether malignancy-related prediction scores (PS) were associated with pathological response to neoadjuvant therapy in breast cancer. **Methods:** Support vector machine classifiers were developed for 1, 3, 5, and 7 mm peritumoral regions using 1716 pathologically confirmed lesions. We assessed PS against postoperative Miller–Payne (MP) grade in 132 patients who completed neoadjuvant therapy and surgery. Ordinal logistic regression examined overall associations, interactions with hormone receptor (HR) status and Ki67, and treatment-stratified associations adjusted for both. Holm correction addressed multiple testing. The 7 mm classifier was refitted using 1584 patients outside the response cohort while retaining the originally selected features. **Results:** Higher 7 mm PS was associated with lower MP grade overall (odds ratio [OR] per 0.1-unit increase, 0.79; 95% confidence interval [CI], 0.66–0.95; Holm-adjusted *p* = 0.048). HR and Ki67 interactions remained significant at 1 mm (adjusted *p* = 0.049 and 0.036, respectively). In 60 complete cases receiving chemotherapy plus anti-HER2 therapy, the original 7 mm PS was associated with MP grade after adjustment (OR, 0.60; 95% CI, 0.42–0.85; adjusted *p* = 0.037). The refitted PS showed an attenuated association in these patients (OR, 0.709; 95% CI, 0.526–0.955; raw *p* = 0.0239; Holm-adjusted *p* = 0.0478). Associations in the original classifier test subsets (*n* = 22–26) were not significant after correction. **Conclusions:** The 7 mm score may complement HR and Ki67 for response assessment during chemotherapy plus anti-HER2 therapy. Interactions at 1 mm suggest variation with molecular characteristics. These findings warrant independent validation.

## 1. Introduction

Breast cancer is one of the most common malignancies in women. Neoadjuvant therapy, including chemotherapy alone and chemotherapy combined with anti-HER2 targeted therapy, is widely used for locally advanced and selected high-risk early breast cancers to downstage tumors and increase opportunities for breast-conserving surgery [1,2]. Nevertheless, breast cancer is highly heterogeneous [3], and pathological responses vary substantially even when treatment is selected according to HER2, hormone receptor (HR), and Ki67 status [4,5]. A noninvasive and reproducible method for predicting response before surgery could therefore help inform treatment selection, identify patients likely to benefit, and reduce ineffective treatment. Ultrasonography is widely used in breast cancer care because it is accessible, real-time, inexpensive, and repeatable. Ultrasound radiomics and artificial intelligence (AI) offer opportunities to predict response to neoadjuvant therapy. These approaches extract quantitative features that are difficult to assess visually and have been investigated for predicting molecular subtype, lymph node status, and treatment response [6,7]. Most imaging studies, however, have focused on the tumor itself, with less attention to its surrounding microenvironment. The peritumoral region includes the invasive interface, reactive stroma, vessels, immune cells, and adjacent normal tissue. Interactions among these components can influence tumor behavior, treatment sensitivity, and resistance [8]. Stromal organization may also affect the distribution of therapeutic antibodies [9]. Peritumoral tissue is spatially heterogeneous: regions at different distances from the tumor may capture the invasive front, reactive stroma, vascular or lymphatic remodeling, and the transition to normal tissue. Studies using PET/CT and contrast-enhanced spectral mammography have examined peritumoral radiomics for evaluating response to neoadjuvant chemotherapy [10,11]. These observations provide a rationale for investigating whether ultrasound patterns around a tumor contain information relevant to treatment response. Assessing multiple spatial scales may therefore help characterize the relationship between peritumoral imaging phenotypes and pathological response.

Molecular characteristics and treatment regimen are important contexts for interpreting these imaging associations. Human epidermal growth factor receptor 2 (HER2) status guides the use of anti-HER2 therapy [12], hormone receptor (HR) status indicates estrogen and/or progesterone receptor expression, and Ki67 provides a measure of proliferative activity [13]. The relationship between an imaging score and response may therefore vary across these characteristics and treatments. The Miller–Payne (MP) system assesses pathological response by comparing tumor cellularity before and after therapy [14]. Its ordered grades allow investigation of the degree of response across a spectrum of tumor regression.

In this study, we examined whether continuous prediction scores (PS) from benign–malignant ultrasound classifiers were associated with MP grade among patients with confirmed breast cancer. Our hypothesis was that malignancy-related peritumoral image patterns might reflect tissue organization and tumor–host interactions that are also relevant to treatment sensitivity. A malignancy score does not itself measure chemosensitivity or resistance; its association with response must be established empirically within the cancer population. We therefore evaluated PS from 1, 3, 5, and 7 mm peritumoral regions, explored variation in the PS–MP relationship by HER2, HR, and Ki67, and assessed associations within treatment groups. These analyses aimed to identify candidate imaging markers for further validation.

## 2. Materials and Methods

### 2.1. Study Design and Study Population

This retrospective study used a two-stage analytical strategy. In the first stage, multiscale peritumoral models were developed and evaluated for the classification of pathologically confirmed breast lesions as benign/borderline or malignant. Continuous model outputs from the 1, 3, 5, and 7 mm peritumoral regions were defined as PS. In the second stage, associations between these scores and pathological response were examined in patients with breast cancer who completed neoadjuvant therapy and surgery and had postoperative MP grades available.

The study was approved by the Medical Ethics Committee of the Second Affiliated Hospital of Xi’an Jiaotong University (approval no. 2023430). The requirement for informed consent was waived because of the retrospective design and de-identification of patient information.

### 2.2. Ultrasound Acquisition and ROI Delineation

For the first stage, breast lesions confirmed by core needle biopsy or surgical pathology were retrospectively collected from the Second Affiliated Hospital of Xi’an Jiaotong University and Shangluo Central Hospital between January 2021 and December 2024. Ultrasound images were acquired using a Siemens Sequoia 512 system with an 18L6 linear-array transducer (4.6–17.8 MHz). Exclusion criteria were treatment with chemotherapy, endocrine therapy, immunotherapy, or other antitumor therapy before ultrasonography; poor image quality; or loss of more than 50% of the intended peritumoral region because of an insufficient field of view.

The model-development cohort comprised 1716 patients, each contributing one breast lesion and one two-dimensional ultrasound image. Of these patients, 706 were from the Second Affiliated Hospital of Xi’an Jiaotong University and 1010 from Shangluo Central Hospital. Pathology identified 750 malignant and 966 benign or borderline lesions. All images were uploaded to the Darwin research platform for model development, evaluation, and PS extraction.

On conventional two-dimensional static ultrasound images, 3 physicians with 5 years of experience in breast ultrasonography manually delineated the visible tumor boundary to define the intratumoral region of interest (ROI). The contours were reviewed and finalized by 1 senior physician with more than 10 years of experience in breast ultrasonography. All physicians involved in delineation and review were blinded to the pathological results.

**Figure 1:**
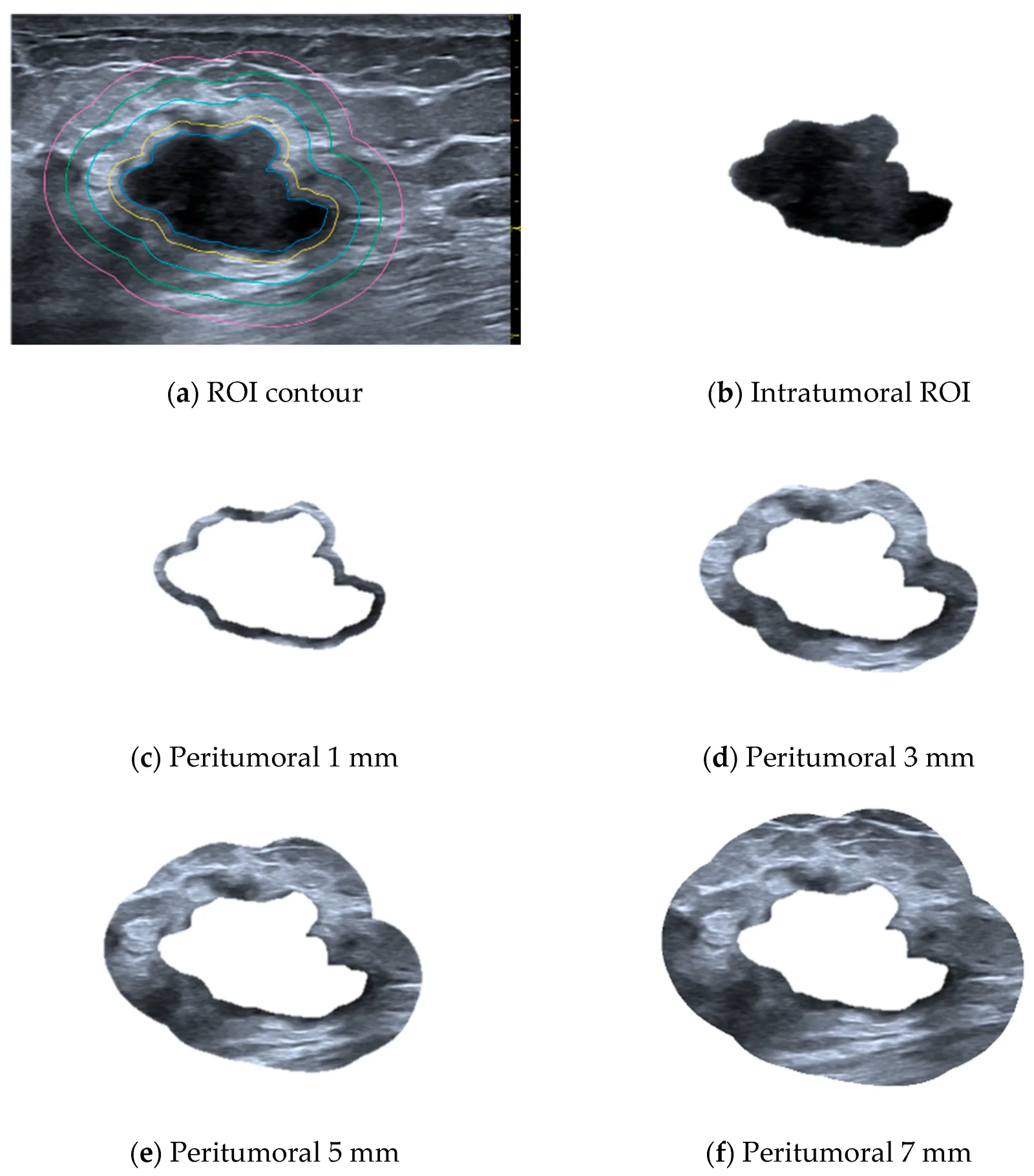
(**a**) The manually delineated tumor boundary is shown in blue. Boundaries expanded outward by 1, 3, 5, and 7 mm are shown in yellow, cyan, green, and pink, respectively. (**b**) Extracted intratumoral ROI. (**c**–**f**) Peritumoral ROIs spanning 0–1, 0–3, 0–5, and 0–7 mm from the tumor boundary, respectively, obtained by subtracting the intratumoral ROI from the corresponding expanded region. These are nested cumulative regions, all excluding the tumor interior. ROI, region of interest.

Using the platform’s margin-expansion function, the tumor ROI was expanded outward by 1, 3, 5, and 7 mm. Each peritumoral ROI was obtained by subtracting the original intratumoral ROI from the corresponding expanded region. The resulting 0–1, 0–3, 0–5, and 0–7 mm regions were nested cumulative regions, all excluding the tumor interior (Figure 1).

### 2.3. Machine Learning and PS Extraction

Radiomic analysis was performed using the PyRadiomics-based workflow integrated into the DARWIN platform (Yizhun AI, Beijing, China; PyRadiomics version: 3.0.1). Before feature extraction, pretreatment two-dimensional ultrasound images were resampled in-plane to a pixel spacing of 0.5 × 0.5 mm using cubic B-spline interpolation. The corresponding region-of-interest (ROI) masks were resampled using nearest-neighbor interpolation. Cumulative peritumoral ROIs were generated by a 2-dimensional Euclidean distance transform outward from the manually delineated tumor boundary, extending 1, 3, 5, and 7 mm, with the intratumoral region excluded from all peritumoral masks.

For each patient, image intensity normalization was performed as a custom preprocessing step implemented by the DARWIN platform, rather than using the native built-in normalization function of PyRadiomics. Mean and standard deviation were calculated from the cumulative 0–7 mm peritumoral region after resampling, excluding the manually delineated tumor ROI. The same patient-specific normalization parameters were used for all four peritumoral scales. The resulting z-scores were clipped to the interval [−3, 3], and gray-level discretization used a fixed bin width of 1. This peritumoral-region-based normalization strategy was intended to reduce variation in image brightness associated with acquisition settings and global brightness variation across ultrasound images by normalizing intensities relative to the local peritumoral tissue rather than the entire field of view.

Radiomic features included first-order intensity statistics, two-dimensional shape descriptors, and texture features derived from the gray-level co-occurrence matrix, gray-level run-length matrix, gray-level size-zone matrix, gray-level dependence matrix, and neighboring gray-tone difference matrix. The exported feature sets included features from the original images and from images processed using wavelet (2 decomposition levels), Laplacian-of-Gaussian (σ = 1.0, 2.0, 3.0 mm), gradient, two-dimensional local binary pattern (radius = 1, 8 neighbors), logarithmic, square, square-root, and exponential transformations. Shape descriptors were calculated from the corresponding ROI masks. Feature definitions and calculations followed the PyRadiomics implementation. The custom intensity normalization step (patient-specific z-score derived from the cumulative peritumoral mask) was not part of native PyRadiomics/IBSI default pipelines and was explicitly defined herein.

The four peritumoral classifiers were developed separately using the same feature-standardization, feature-selection, and classification framework. For each scale, feature-standardization parameters were estimated exclusively from the corresponding classifier-training set and subsequently applied unchanged to the test data. Model-based feature selection was performed within the training set using an L1-penalized logistic-regression selector (LASSO). The selector was configured with three-fold cross-validation and accuracy as the evaluation metric, with the maximum number of retained features capped at 50% of the input features. The selected features were then used to train a support vector machine (SVM) classifier with a radial basis function (RBF) kernel for benign–malignant classification. The RBF hyperparameters, C and gamma, were tuned using grid-search cross-validation within the training set over C ∈ {0.01, 0.1, 1, 10, 100} and γ ∈ {0.001, 0.01, 0.1, 1}. Balanced class weighting was not enabled.

Each classifier generated a continuous prediction score (PS), with higher values indicating a stronger model-assessed tendency toward malignancy. These malignancy-related scores were subsequently used to examine associations with pathological response to neoadjuvant therapy. Postoperative Miller–Payne (MP) grade was not used for feature selection or classifier training.

Among the 132 patients in the response cohort, 106, 110, 109, and 107 had been assigned to the training sets of the original 1, 3, 5, and 7 mm malignancy classifiers, respectively; the remaining 26, 22, 23, and 25 had been assigned to their respective test sets. Training and test membership differed among scales. MP grade was not used for feature selection or classifier training, but the response cohort was not held out from the original classifier-development pipeline.

To assess sensitivity of the 7 mm association to patient overlap during classifier fitting, we removed all records for the 132 response-cohort patients by patient ID from the available exported 7 mm feature matrix, leaving a total of 1584 unique patients. We retained the feature set selected in the original analysis; image preprocessing, feature extraction, and LASSO feature selection were not repeated. Feature standardization was fitted within the development data. An RBF-kernel SVM was refitted, with C and gamma selected by 5-fold stratified cross-validation using the benign–malignant ROC AUC. The selected settings were C = 1 and gamma = 0.01. Sigmoid probability calibration also used only the remaining development patients with 5-fold cross-validation. The calibrated classifier generated new 7 mm PS values for the 132 response-cohort patients. This sensitivity analysis removed these patients from SVM fitting, tuning, standardization, and calibration, but the retained features had been selected in the original analysis; it did not independently validate the complete feature-selection and classification pipeline.

### 2.4. Clinical and Pathological Data and Response Assessment

For the second stage, patients who received neoadjuvant therapy and had postoperative pathological response assessments were selected from the 750 malignant cases. Twenty-two cases with non-epithelial malignancies or rare special histological types, including primary breast fibrosarcoma, malignant phyllodes tumor, lymphoma, neuroendocrine carcinoma, and myoepithelial carcinoma, were excluded. Among the remaining 728 breast cancer cases, 332 were excluded because complete clinical or pathological records could not be obtained, leaving 396 potentially eligible patients.

**Figure 2:**
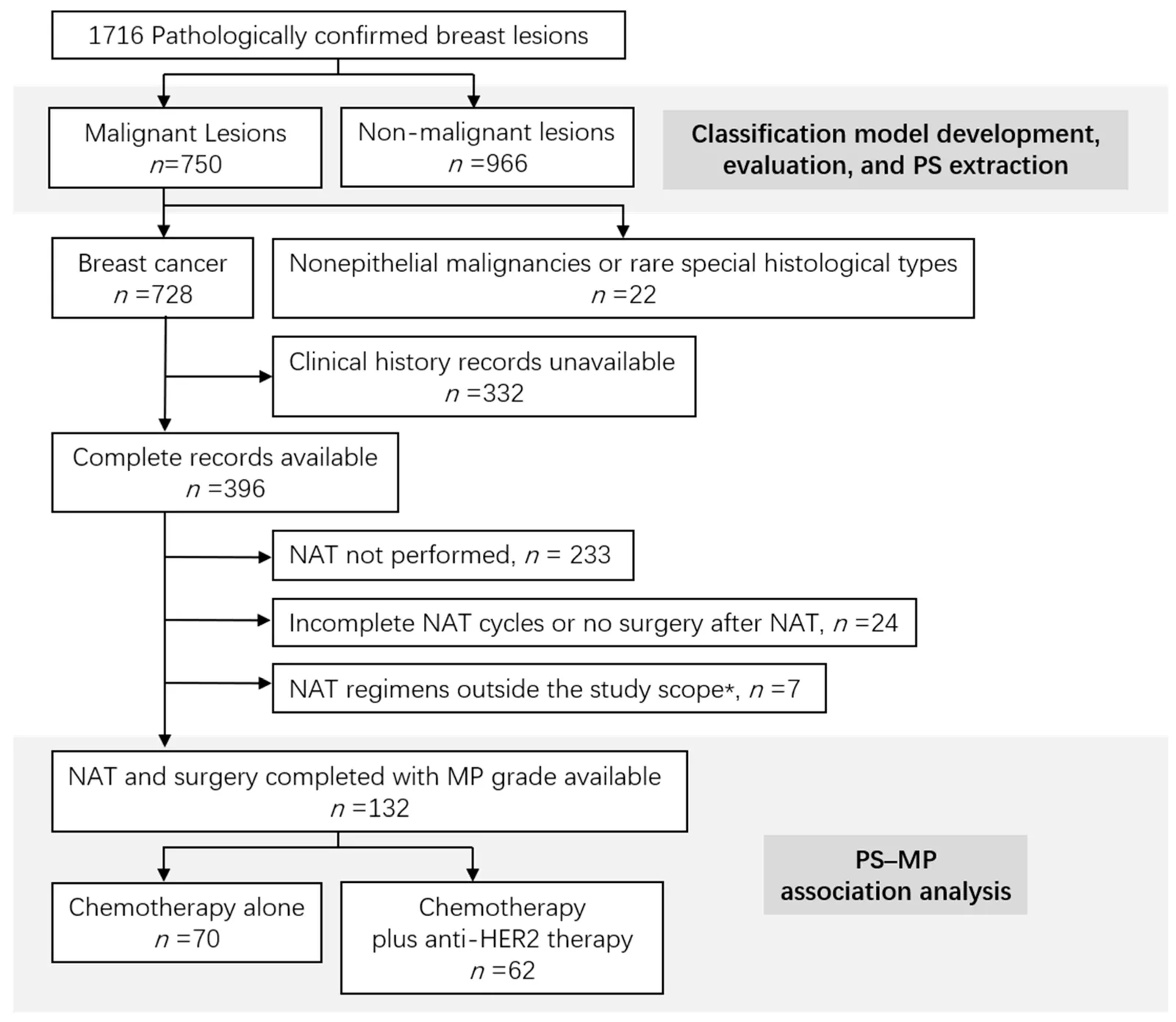
Study flow diagram. The model-development cohort and the subset included in the PS–MP association analyses are shown. * Regimens outside the study scope included immunotherapy, anti-VEGFR therapy, and CDK4/6 inhibitors. NAT, neoadjuvant therapy; PS, prediction score; MP, Miller–Payne grade; HER2, human epidermal growth factor receptor 2.

Further exclusions were upfront surgery without neoadjuvant therapy (*n* = 233), incomplete neoadjuvant therapy cycles or no surgery after neoadjuvant therapy (*n* = 24), and treatment regimens outside the prespecified study scope, including immunotherapy, anti-VEGFR therapy, or CDK4/6 inhibitors (*n* = 7). The final response-analysis cohort comprised 132 patients with primary invasive breast cancer who completed neoadjuvant therapy and surgery and had postoperative MP grades: 70 received chemotherapy alone and 62 received chemotherapy plus anti-HER2 targeted therapy (Figure 2). Clinical and pathological data included age, tumor size, neoadjuvant treatment regimen, HER2 expression, hormone receptor (HR) status, Ki67 index, and postoperative MP grade. HER2, HR, and Ki67 were assessed in pretreatment specimens. HER2 immunohistochemical (IHC) scores were classified as 0, 1+, 2+, or 3+. HER2 positivity was defined as IHC 3+ or IHC 2+ with positive fluorescence in situ hybridization (FISH); IHC 0 or 1+, or IHC 2+ with negative FISH, was considered HER2-negative.

Estrogen receptor (ER) or progesterone receptor (PR) positivity was defined as nuclear staining in at least 1% of tumor cells. HR-positive status required positivity for either ER or PR; tumors negative for both were HR-negative. Ki67 was recorded as a percentage and treated as a continuous variable in the main analyses. A threshold of 30% was used for supplementary analyses comparing Ki67 < 30% with Ki67 ≥ 30% [13]. Pathological response was assessed using postoperative MP grade, ranging from 1 to 5, with higher grades indicating greater loss of tumor cellularity and a better response. Grade 1 indicated no appreciable reduction in tumor cells; grade 2, a reduction of up to 30%; grade 3, a reduction of 30–90%; grade 4, a reduction of more than 90%; and grade 5, no residual invasive carcinoma in the primary tumor site.

### 2.5. Statistical Analysis

Analyses were performed using R version 4.5.2. Continuous variables were assessed for normality using the Shapiro–Wilk test and summarized as mean ± standard deviation or median with the first and third quartiles (Q1–Q3), as appropriate. Categorical variables were summarized as counts and percentages. Available baseline data were compared between the 396 patients with accessible complete records and the 332 patients without accessible complete records to examine potential differences at the record-retrieval stage. Comparisons of age, ordered age groups, and tumor-size categories were performed using Mantel–Haenszel tests. The 132-patient response cohort was presented for descriptive context. Differences in PS across the four scales were assessed using the Friedman test, followed by six paired Wilcoxon signed-rank comparisons with Bonferroni correction.

Separate univariable ordinal logistic regression models were fitted for each scale to assess the association between PS and MP grade in the full response cohort. MP was treated as an ordered outcome from 1 to 5. PS effects were expressed as odds ratios (ORs) with 95% confidence intervals (CIs) per 0.1-unit increase; an OR below 1 indicated lower odds of a higher MP grade.

Sensitivity analyses repeated the univariable ordinal logistic regression separately among response-cohort patients assigned to the test set of the corresponding malignancy classifier. These subsets comprised 26, 22, 23, and 25 patients for the 1, 3, 5, and 7 mm models, respectively. Patient IDs were matched to the original training/test assignments, and each analysis used the PS from its corresponding classifier. Because patient membership differed across scales, the subset estimates were interpreted separately, with emphasis on effect sizes and uncertainty. Holm correction was applied across the four test-subset association tests.

Exploratory Spearman rank correlations between PS and MP grade were calculated within HER2 IHC strata (0, 1+, 2+, and 3+) and HR strata (negative and positive). Separate ordinal logistic models included PS, HR or continuous Ki67, and their interaction to examine effect modification. HR-negative status was the reference category, and continuous Ki67 was scaled per 10-percentage-point increase. Supplementary analyses examined PS-by-Ki67-group interactions and fitted separate ordinal logistic models within the Ki67 <30% and ≥30% groups.

For the continuous Ki67 interaction models, the predicted probability of MP grade ≥4 was plotted across the observed PS range at Ki67 values of 20%, 40%, and 70%, corresponding to Q1, the median, and Q3. Pointwise 95% Wald CIs were calculated on the logit scale using the joint covariance matrix of the regression coefficients and the relevant MP threshold, then transformed back to the probability scale.

Multivariable ordinal logistic regression was performed within each treatment group, entering PS from one scale at a time and adjusting for HR status and continuous Ki67. Analyses involving Ki67 used complete cases without imputation. Ki67 was missing in one patient receiving chemotherapy alone and two receiving chemotherapy plus anti-HER2 therapy, leaving 69 and 60 patients, respectively, for the treatment-stratified adjusted analyses.

An exploratory binary response analysis was conducted in the same 60 complete cases receiving chemotherapy plus anti-HER2 therapy. High response was defined as MP grades 4–5 and low response as grades 1–3. Three logistic models were fitted: a 7 mm PS model, a clinical model containing HR and Ki67, and a combined model containing all three predictors. Receiver operating characteristic (ROC) curves and apparent areas under the curve (AUCs) were calculated from the fitted response probabilities. Nominal 95% CIs for the apparent AUCs were calculated using the DeLong method in pROC.

Likelihood ratio tests compared the combined model with each nested model fitted to the same patients. The comparison with the clinical model assessed the contribution of adding 7 mm PS (1 degree of freedom), and the comparison with the PS model assessed the joint contribution of adding HR and Ki67 (2 degrees of freedom). Test statistics were calculated as twice the difference in maximized log-likelihoods, with *p* values obtained from the corresponding asymptotic chi-square distribution. These tests evaluated improvement in model fit.

Internal validation of the three binary logistic models used 1000 ordinary patient-level bootstrap samples of 60 patients drawn with replacement (random seed, 20261014). The same resampled patients were used for all three models in each iteration. Each model was refitted in the bootstrap sample and evaluated both in that sample and in the original 60 patients. Optimism was calculated as the bootstrap-sample AUC minus the AUC of the bootstrap-fitted model in the original sample, and the optimism-corrected AUC was obtained by subtracting the mean optimism from the original apparent AUC. Convergence, model rank, and complete or quasi-complete separation were checked. Four resamples produced separation in the clinical and combined models; these resamples were excluded from all three models’ optimism summaries, leaving 996 common valid resamples. Failed resamples were not replaced. Corrected AUCs were reported as point estimates. This validation assessed the downstream logistic models conditional on the existing PS and the selected 7 mm scale.

Multiplicity control was introduced during revision, with tests grouped by scientific question. Holm correction was applied separately to six families: four overall PS–MP associations; four test-subset PS–MP associations; 12 interaction tests involving HR, continuous Ki67, or dichotomized Ki67 across four scales; 32 molecular subgroup tests comprising 16 HER2-stratified correlations, eight HR-stratified correlations, and eight Ki67-stratified ordinal regression associations; eight treatment-stratified adjusted PS associations; and two likelihood ratio comparisons. HR and Ki67 main-effect coefficients in the treatment-stratified models were reported as adjustment terms and were not included in the eight-test PS family.

For the classifier-refitting sensitivity analysis, we replaced the original 7 mm PS with the refitted 7 mm PS and repeated the treatment-stratified multivariable ordinal logistic models in the same complete-case samples (69 receiving chemotherapy alone and 60 receiving chemotherapy plus anti-HER2 therapy). Each model adjusted for HR status and continuous Ki67. PS odds ratios were expressed per 0.1-unit increase, with nominal 95% confidence intervals. The two treatment-specific refitted-PS coefficient tests formed an additional exploratory family for Holm correction; HR and Ki67 coefficients were reported as adjustment terms.

Statistical significance within these families was assessed using Holm-adjusted *p* < 0.05. Both raw and adjusted *p* values were reported in the relevant tables. Tests of regression coefficients and correlations were two-sided. Reported 95% CIs were nominal intervals, and the plotted probability bands were pointwise rather than simultaneous intervals.

## 3. Results

### 3.1. Diagnostic Performance of the Peritumoral Classifiers

**Figure 3:**
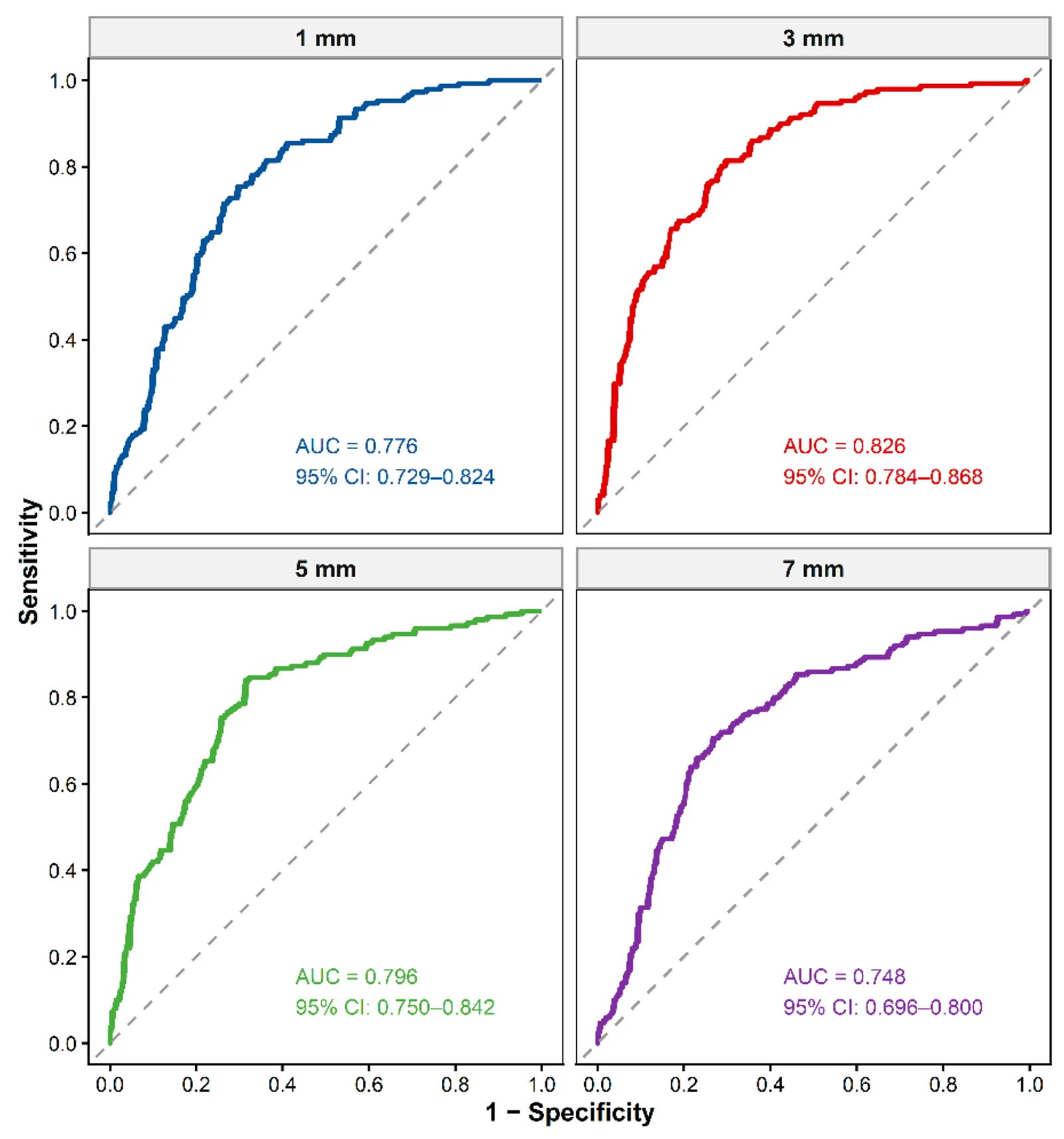
ROC curves of multiscale peritumoral ultrasound classifiers for distinguishing malignant from benign/borderline breast lesions. The panels show results for the 1, 3, 5, and 7 mm models in their respective test sets. AUCs were 0.776 (95% CI, 0.729–0.824), 0.826 (95% CI, 0.784–0.868), 0.796 (95% CI, 0.750–0.842), and 0.748 (95% CI, 0.696–0.800), respectively. The horizontal axis is 1 − specificity, and the vertical axis is sensitivity. The gray dashed diagonal indicates no discrimination (AUC = 0.5). ROC, receiver operating characteristic; AUC, area under the curve; CI, confidence interval.

In their respective test sets, the 1, 3, 5, and 7 mm classifiers distinguished malignant from benign/borderline lesions with AUCs of 0.776 (95% CI, 0.729–0.824), 0.826 (0.784–0.868), 0.796 (0.750–0.842), and 0.748 (0.696–0.800), respectively (Figure 3). The 3 mm classifier had the highest AUC point estimate. All four scores were retained for the subsequent response-association analyses.

### 3.2. Clinical and Pathological Characteristics

The response-analysis cohort comprised 132 patients who completed neoadjuvant therapy and surgery and had postoperative MP grades available (Table 1).

Median age was 50 years (range, 28–73). Tumor size was categorized as ≤10, >10 to ≤20, >20 to ≤50, and >50 mm; The most frequent tumor-size category was >10 to ≤20 mm, comprising 67 patients (50.76%). Seventy patients (53.03%) received chemotherapy alone, and 62 (46.97%) received chemotherapy plus anti-HER2 therapy. HR was positive in 77 patients (58.33%) and negative in 55 (41.67%). HER2 IHC scores of 0, 1+, 2+, and 3+ were recorded in 25 (18.94%), 26 (19.70%), 29 (21.97%), and 52 (39.39%) patients, respectively.

Ki67 was unavailable for three patients: one in the chemotherapy-alone group and two in the chemotherapy plus anti-HER2 group. Among the 129 patients with available data, median Ki67 was 40% (Q1–Q3, 20–70%), and mean Ki67 was 43.2% ± 26.3%. Postoperative MP grades 1, 2, 3, 4, and 5 occurred in 16 (12.12%), 24 (18.18%), 24 (18.18%), 14 (10.61%), and 54 (40.91%) patients, respectively.

To examine differences at the record-retrieval stage, available baseline characteristics were compared between 396 patients with accessible complete records and 332 patients without accessible complete records (Table S1). Median ages were 52 and 53 years, respectively (*p* = 0.110). Neither ordered age categories nor tumor-size categories showed a statistically significant difference between these groups (*p* = 0.457 and *p* = 0.177, respectively).

### 3.3. PS Distributions and Overall Associations with MP Grade

PS from the four peritumoral models were extracted for all 132 patients; their descriptive statistics are presented in Table 2. Their distributions were non-normal, with medians of 0.729, 0.731, 0.774, and 0.751 at 1, 3, 5, and 7 mm, respectively (Table 2). The Friedman test indicated an overall difference across scales (χ^2^ = 14.108, *p* = 0.003). Of six paired comparisons, only the 5 mm PS was higher than the 1 mm PS after Bonferroni correction (adjusted *p* = 0.036). All other adjusted *p* values were ≥0.090. These comparisons describe score distributions and do not establish differences in predictive performance.

**Figure 4:**
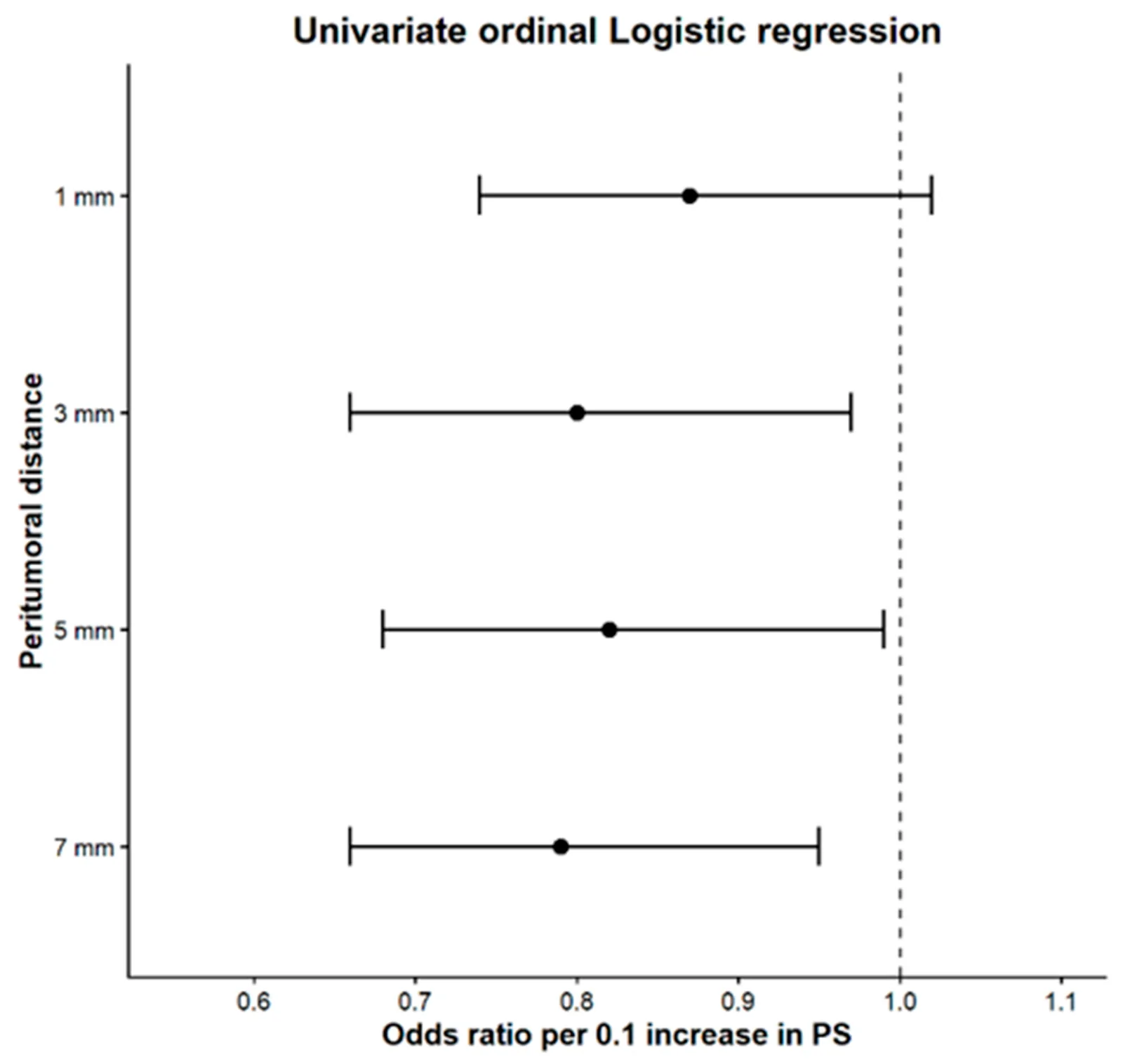
Forest plot of univariable ordinal logistic associations between peritumoral PS and MP grade in 132 patients. Black points indicate ORs per 0.1-unit increase in PS, and horizontal lines indicate 95% CIs. The vertical dashed line indicates OR = 1. Statistical interpretation is based on Holm-adjusted *p* values in Table 3; only the 7 mm association remained significant. The CIs were not adjusted for multiplicity. PS, prediction score; MP, Miller–Payne grade; OR, odds ratio; CI, confidence interval.

In separate univariable ordinal logistic models, OR point estimates were below 1 at all four scales (Table 3 and Figure 4). After Holm correction across the four tests, only the 7 mm association remained statistically significant: the OR per 0.1-unit increase in PS was 0.79 (95% CI, 0.66–0.95; Holm-adjusted *p* = 0.048). The corresponding ORs at 1, 3, and 5 mm were 0.87, 0.80, and 0.82, with Holm-adjusted *p* values of 0.079, 0.066, and 0.074, respectively. In the scale-specific test subsets, none of the PS–MP associations were statistically significant after Holm correction (Table S2).

### 3.4. Molecular Pathological Factors and PS–MP Associations

Exploratory Spearman analyses examined PS–MP associations within HER2 and HR strata. Ordinal logistic interaction models were additionally used to assess effect modification by HR and Ki67.

#### 3.4.1. HER2-Stratified Analysis

**Figure 5:**
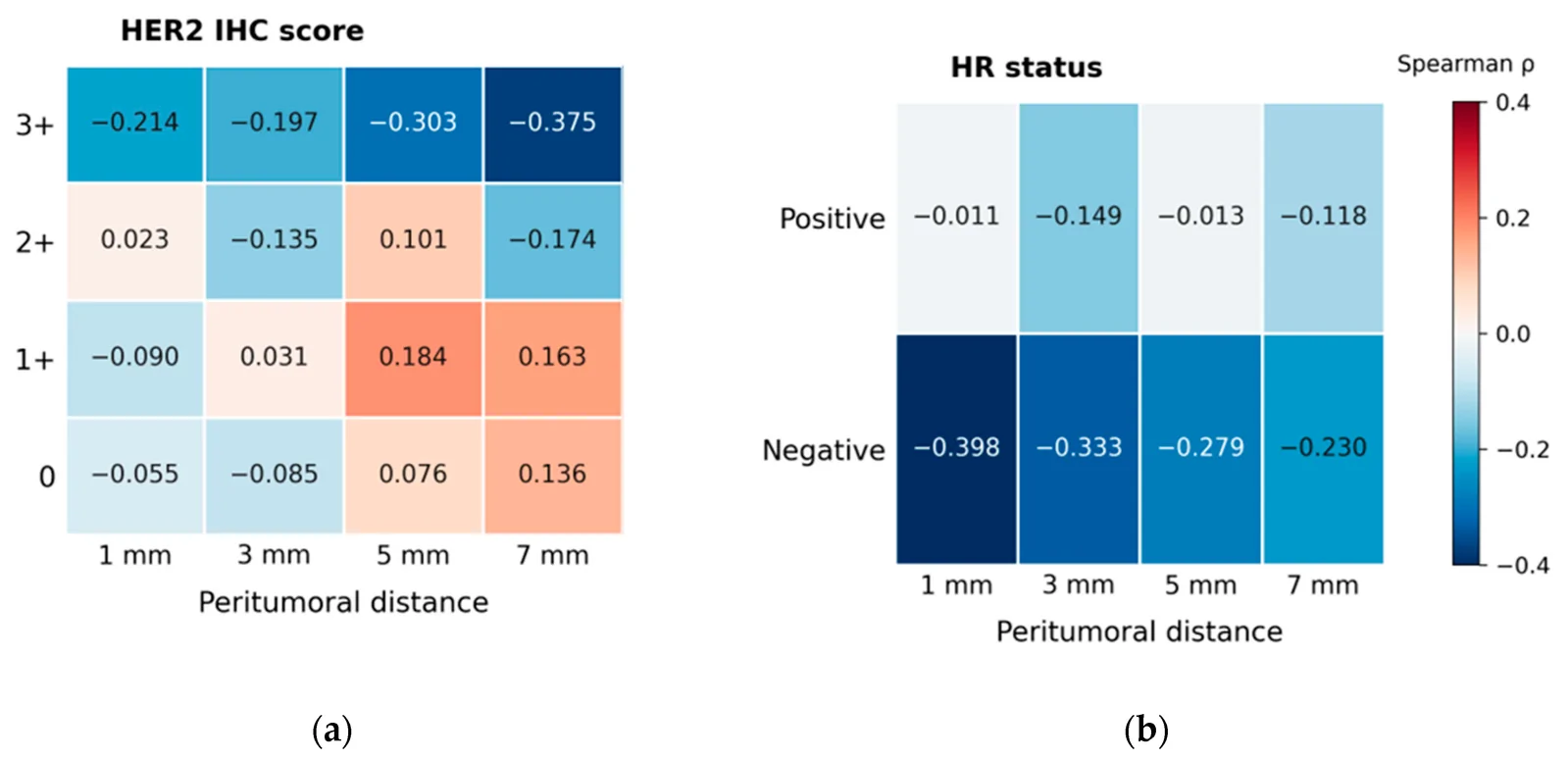
Heatmaps of Spearman correlations between peritumoral PS and MP grade stratified by HER2 and HR status. (**a**) HER2 IHC strata of 0, 1+, 2+, and 3+. (**b**) HR-negative and HR-positive strata. Columns represent peritumoral scales of 1, 3, 5, and 7 mm. Cell values show Spearman ρ. Both panels use the same color scale, with blue indicating negative and red positive correlations. No correlation shown was significant after Holm correction within the 32-test molecular subgroup family; therefore, no significance stars are displayed. Numerical results are provided in Tables S3 and S4. HER2, human epidermal growth factor receptor 2; IHC, immunohistochemistry; HR, hormone receptor; PS, prediction score; MP, Miller–Payne grade.

Within the HER2 IHC 3+ subgroup, the Spearman correlation coefficients at 5 and 7 mm were −0.303 and −0.375, respectively (Holm-adjusted *p* = 0.725 and 0.174). No PS–MP correlation in any HER2 IHC stratum remained statistically significant after correction within the 32-test molecular subgroup family (Figure 5a and Supplementary Table S3).

#### 3.4.2. HR-Stratified and Interaction Analyses

Among HR-negative patients, Spearman correlations at 1, 3, 5, and 7 mm were −0.398, −0.333, −0.279, and −0.230, respectively. None remained statistically significant after Holm correction (adjusted *p* = 0.090, 0.338, 0.936, and 1.000). No significant correlation was observed in the HR-positive group after correction (Figure 5b and Supplementary Table S4).

In ordinal logistic models including PS, HR, and their interaction, the interaction at 1 mm remained statistically significant after Holm correction across the 12 interaction tests (interaction OR, 1.80; 95% CI, 1.20–2.71; adjusted *p* = 0.049). The interaction OR indicates that the PS effect OR was higher in HR-positive than in HR-negative patients. Interactions at 3, 5, and 7 mm were not statistically significant (Table 4).

#### 3.4.3. Interaction and Subgroup Analyses Involving Ki67

**Figure 6:**
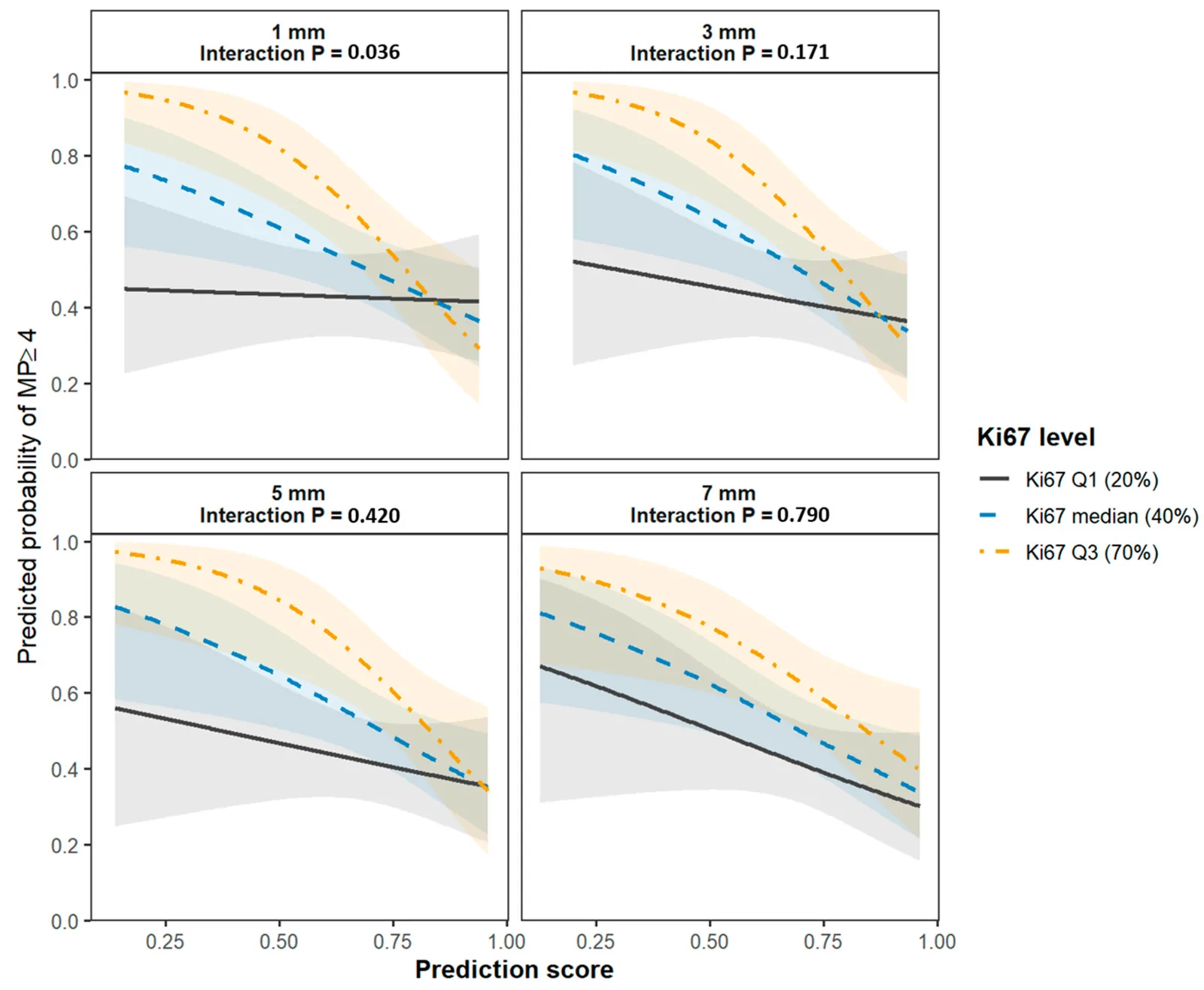
Model-predicted probability of MP grade ≥4 across PS values at different Ki67 levels. Separate ordinal logistic models including PS, continuous Ki67, and their interaction were fitted at each scale in 129 complete cases. Ki67 was fixed at Q1 (20%), the median (40%), and Q3 (70%), shown as gray solid, blue dashed, and orange dot-dashed lines, respectively. Shading shows pointwise 95% CIs, without multiplicity adjustment. Panel labels show interaction *p* values after Holm correction across 12 interaction tests. PS, prediction score; MP, Miller–Payne grade; Q1, first quartile; Q3, third quartile.

The continuous Ki67 interaction analyses included 129 complete cases. After Holm correction, the PS × Ki67 interaction remained statistically significant at 1 mm (interaction OR, 0.90; 95% CI, 0.84–0.97; adjusted *p* = 0.036), but not at 3, 5, or 7 mm (adjusted *p* = 0.171, 0.420, and 0.790, respectively; Table 5). At 1 mm, each 10-percentage-point increase in Ki67 multiplied the PS effect OR by 0.90. Model-predicted probabilities of MP grade ≥4 at Ki67 values of 20%, 40%, and 70%, with pointwise 95% confidence bands, are shown in Figure 6.

Analyses using a 30% Ki67 threshold included 46 patients with Ki67 < 30% and 83 with Ki67 ≥ 30%. The PS × Ki67-group interaction remained significant only at 1 mm (interaction OR, 0.611; 95% CI, 0.438–0.851; Holm-adjusted *p* = 0.044; Table S5). In the Ki67 ≥ 30% group, the 1 mm PS association remained significant after correction within the molecular subgroup family (OR per 0.1-unit increase, 0.67; adjusted *p* = 0.032); the corresponding adjusted *p* values at 3, 5, and 7 mm were 0.055, 0.252, and 0.324. No association was significant in the Ki67 < 30% group (Supplementary Table S6).

### 3.5. Treatment-Stratified Adjusted Associations

Given the close relationship between HER2 status and selection of anti-HER2 therapy, analyses were stratified by the treatment actually received.

Treatment-stratified multivariable ordinal logistic regression was performed in complete cases (69 patients receiving chemotherapy alone and 60 receiving chemotherapy plus anti-HER2 therapy). Within each treatment group, four ordinal logistic models were fitted; each incorporated PS from one spatial scale and adjusted for HR status and continuous Ki67. HER2 was not additionally entered into these treatment-stratified models.

**Figure 7:**
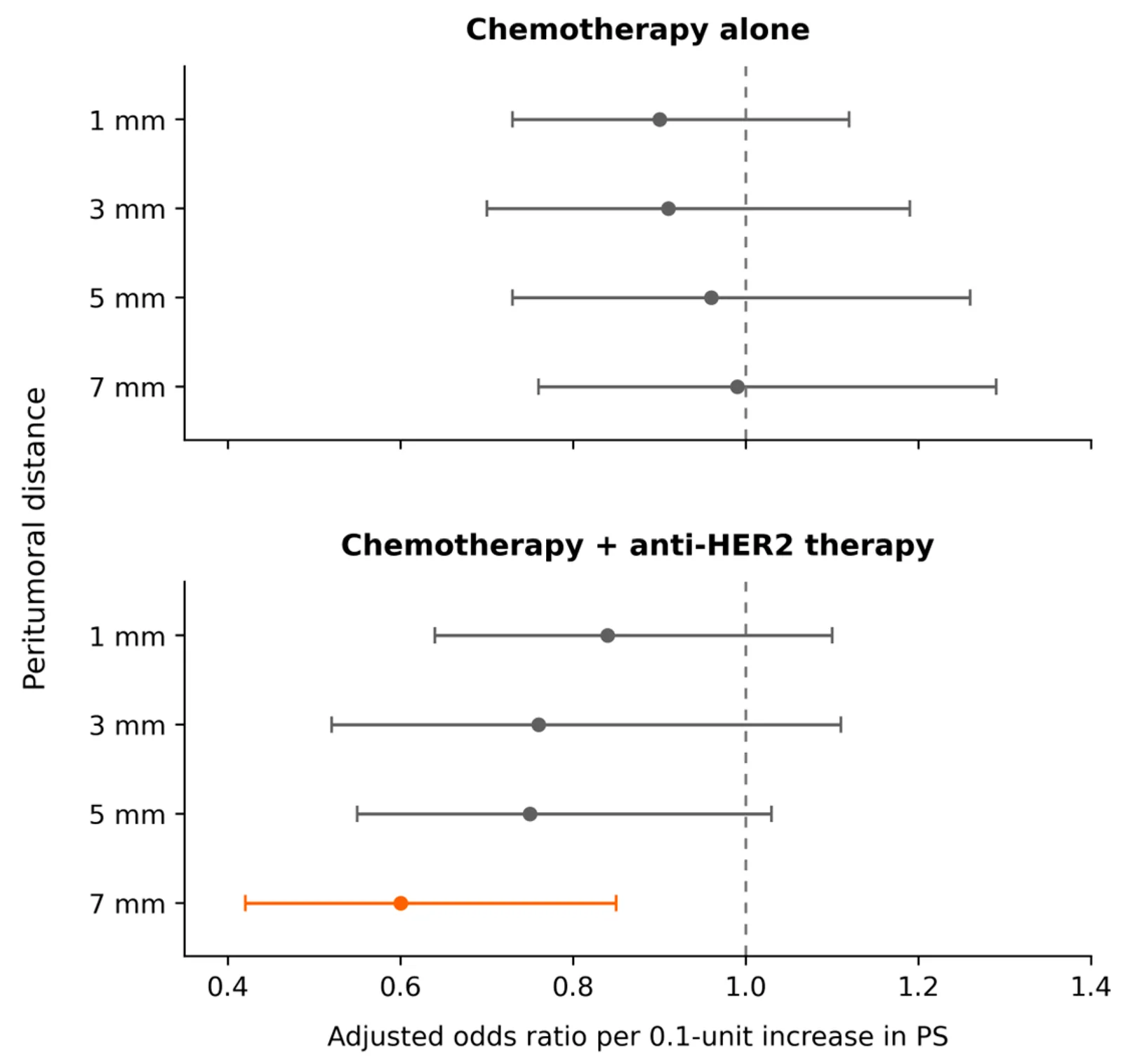
Treatment-stratified forest plots of adjusted associations between peritumoral PS and MP grade. The upper panel shows chemotherapy alone (*n* = 69), and the lower panel shows chemotherapy plus anti-HER2 therapy (*n* = 60). Separate models at each scale adjusted for HR and continuous Ki67. Points indicate adjusted ORs per 0.1-unit increase in PS; horizontal lines indicate 95% CIs and the vertical dashed line indicates OR = 1. Orange indicates a Holm-adjusted *p* value < 0.05 across the eight treatment-stratified PS tests. PS, prediction score; MP, Miller–Payne grade; HR, hormone receptor; HER2, human epidermal growth factor receptor 2; OR, odds ratio; CI, confidence interval.

In the chemotherapy-alone group, adjusted PS ORs ranged from 0.90 to 0.99, and no PS association was significant after Holm correction across the eight treatment-stratified PS tests (all adjusted *p* = 1.000; Table 6). In the chemotherapy plus anti-HER2 group, higher 7 mm PS remained associated with lower odds of a higher MP grade after adjustment for HR and Ki67 and multiplicity correction (OR per 0.1-unit increase, 0.60; 95% CI, 0.42–0.85; Holm-adjusted *p* = 0.037). Associations at 1, 3, and 5 mm were not statistically significant (adjusted *p* = 1.000, 0.960, and 0.560, respectively; Figure 7).

### 3.6. Exploratory Binary Response Models and Internal Validation

The exploratory binary response analysis included the same 60 complete cases receiving chemotherapy plus anti-HER2 therapy: 17 had MP grades 1–3, and 43 had MP grades 4–5. Adding 7 mm PS to the clinical model containing HR and Ki67 improved model fit (likelihood ratio χ^2^ = 6.799, df = 1; Holm-adjusted *p* = 0.01824). Adding HR and Ki67 jointly to the 7 mm PS model did not significantly improve fit (χ^2^ = 2.891, df = 2; adjusted *p* = 0.23568; Table 7).

**Figure 8:**
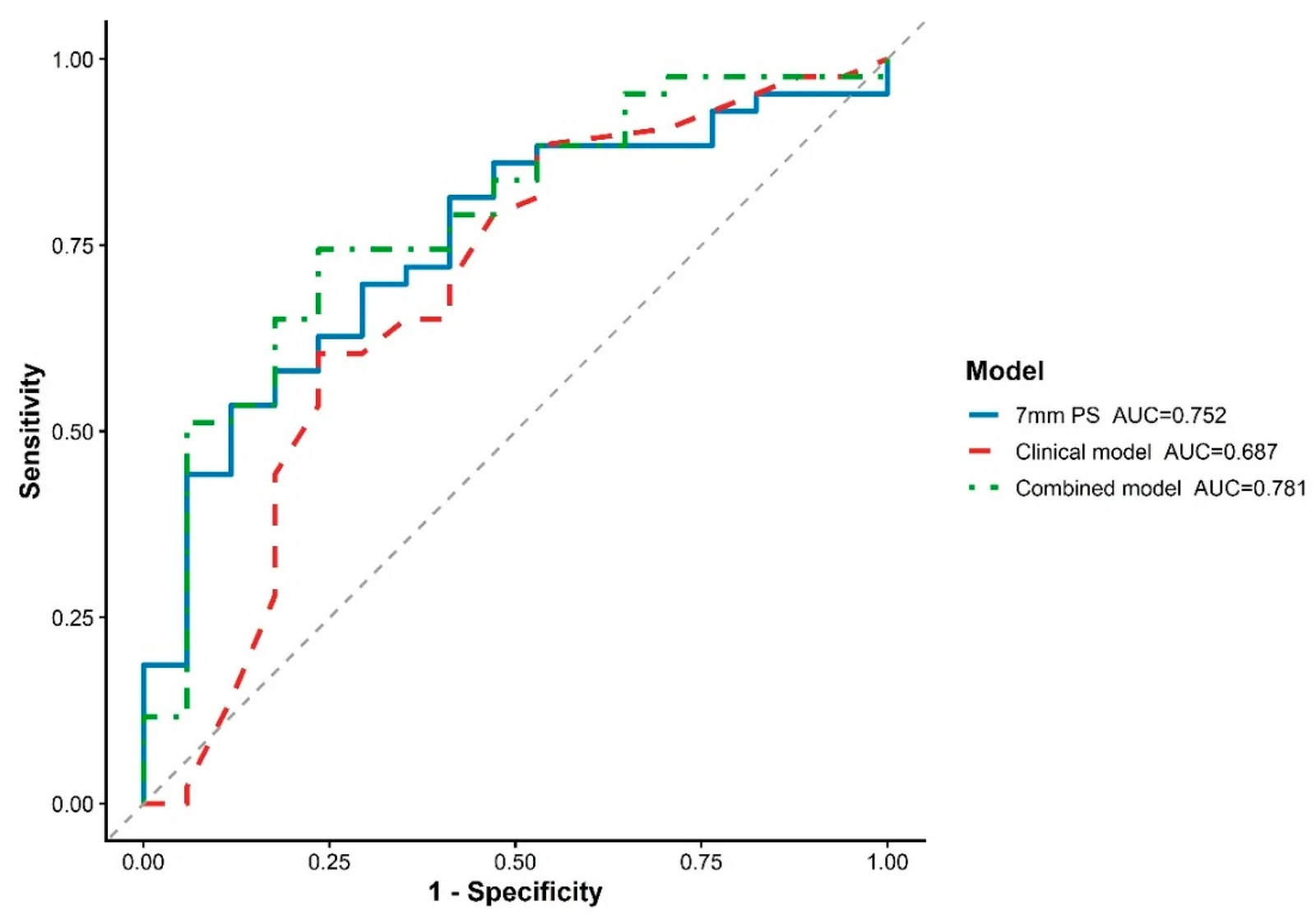
Apparent ROC curves for high pathological response in 60 patients receiving chemotherapy plus anti-HER2 therapy. High response was defined as MP grades 4–5 (*n* = 43) and low response as grades 1–3 (*n* = 17). The blue solid line shows the 7 mm PS model, the red dashed line the clinical model including HR and Ki67, and the green dot-dashed line the combined model. Curves and legend AUCs were obtained from models fitted and evaluated in the same patients. They represent apparent discrimination; optimism-corrected AUCs are reported separately in Table S7. The gray diagonal indicates no discrimination. ROC, receiver operating characteristic; AUC, area under the curve; PS, prediction score; HR, hormone receptor; MP, Miller–Payne grade; HER2, human epidermal growth factor receptor 2.

Apparent AUCs were 0.752 (95% CI, 0.620–0.885) for the 7 mm PS model, 0.687 (0.521–0.854) for the clinical model, and 0.781 (0.650–0.912) for the combined model (Figure 8 and Table S7). Of 1000 bootstrap resamples, four produced complete or quasi-complete separation in the clinical and combined models, leaving 996 common valid resamples for the optimism summaries. The optimism-corrected AUCs were 0.758, 0.649, and 0.742, respectively. Thus, the combined model had the highest apparent AUC point estimate, whereas the 7 mm PS model had the highest optimism-corrected point estimate.

### 3.7. Classifier-Refitting Sensitivity Analysis

In the classifier-refitting sensitivity analysis, a higher refitted 7 mm PS was associated with lower odds of a higher MP grade in the chemotherapy plus anti-HER2 therapy group after adjustment for HR status and continuous Ki67 (*n* = 60; adjusted OR per 0.1-unit increase, 0.709; nominal 95% CI, 0.526–0.955; raw *p* = 0.0239; Holm-adjusted *p* = 0.0478). The association was attenuated compared with that obtained using the original PS. No significant association was observed in the chemotherapy-alone group (*n* = 69; adjusted OR, 0.938; nominal 95% CI, 0.743–1.184; raw and Holm-adjusted *p* = 0.5896; Table 8).

## 4. Discussion

This study investigated whether malignancy-related scores derived from peritumoral ultrasound also contain information about pathological response to neoadjuvant therapy. After Holm correction, higher 7 mm PS remained associated with lower MP grade in the overall response cohort and, after adjustment for HR and Ki67, among patients receiving chemotherapy plus anti-HER2 therapy. Interactions with HR status and continuous Ki67 remained significant at 1 mm. A separate 7 mm classifier refit excluding the response cohort from SVM fitting produced an attenuated association in the chemotherapy plus anti-HER2 group. The binary response analysis further indicated that adding 7 mm PS to HR and Ki67 improved model fit. Together, these findings support investigating peritumoral PS as a source of response-related information that may complement the molecular markers examined. Recent imaging studies provide a broader context for these findings. You et al. developed an ultrasound deep learning framework incorporating clinical information and demonstrated its ability to predict pathological complete response (pCR) in an external validation cohort [15]. Zhang et al. investigated multimodal imaging for HER2 assessment and developed additional models for neoadjuvant treatment response, illustrating the potential relationship between imaging-derived molecular phenotypes and treatment outcomes [16]. These studies support evaluating imaging alongside established pathological markers. Our study examines a complementary question: whether variation in an existing malignancy-related imaging score is associated with the degree of pathological response measured by ordered MP grade. Evidence specifically concerning peritumoral ultrasound is also emerging. Yao et al. evaluated pretreatment radiomics from a 3 mm peritumoral region for pCR prediction; the best-performing peritumoral model had lower discrimination than the corresponding intratumoral model in a temporal test cohort [17]. In a prospective study of 30 patients, Dolui et al. incorporated a 5 mm peritumoral region into multiparametric ultrasound analysis and reported contributions from both baseline peritumoral information and early-treatment measurements [18]. Putin et al. combined serial quantitative ultrasound, shear-wave elastography, and clinical information to distinguish residual cancer burden (RCB) classes 0/I from II/III in 135 patients, using internal cross-validation [19]. Differences in imaging methods, response endpoints, and validation designs preclude direct performance comparisons with our association analyses. MP grade, pCR, and RCB capture related but distinct aspects of pathological response.

The association between higher PS and poorer pathological response in this study suggests that some malignancy-related imaging patterns may also be relevant to treatment sensitivity. PS summarizes an imaging phenotype learned for benign–malignant discrimination; it does not directly quantify histological grade or drug resistance. Restricting the response cohort to confirmed cancers narrows the disease spectrum; diagnostic discrimination in the original cohort therefore cannot establish response prediction within this subgroup. Nevertheless, continuous differences among malignant lesions may reflect variations in stromal organization, image heterogeneity, or tumor–host interactions. Hagenaars et al. reported an association between lower stromal content in pretreatment biopsies and higher MP grade in HER2-negative breast cancer [20]. This supports investigating tissue composition as a possible link between imaging and response, while the histological correlates of PS remain to be established. Interactions with HR status and continuous Ki67 remained significant only in the 1 mm model after multiplicity correction (adjusted *p* = 0.049 and 0.036, respectively). The Ki67 interaction indicated a stronger inverse PS–MP relationship as proliferative activity increased. The supplementary analysis using a 30% Ki67 threshold showed a corresponding interaction at 1 mm, although it used the same patients and therefore does not constitute independent replication. The continuous Ki67 interaction at 3 mm and all HER2- and HR-stratified correlations did not remain significant after correction. Microscopic spatial profiling and functional studies have identified fibroblast-associated cellular neighborhoods and interactions among malignant cells, fibroblasts, and immune cells in breast cancer [21], including heterogeneity at the tumor–immune interface in HER2-positive disease [22]. These findings motivate investigation of tissue organization near the tumor margin; ultrasound does not directly resolve the underlying cellular signaling.

In the chemotherapy plus anti-HER2 group, 7 mm PS remained associated with MP grade after adjustment for HR and Ki67 (OR per 0.1-unit increase, 0.60; 95% CI, 0.42–0.85; Holm-adjusted *p* = 0.037). In the classifier-refitting sensitivity analysis, the 7 mm SVM was fitted using 1584 patients outside the response cohort, while retaining the originally selected features. The adjusted association in the same 60 complete cases receiving chemotherapy plus anti-HER2 therapy was attenuated but remained statistically significant within the two-test sensitivity family (OR per 0.1-unit increase, 0.709; nominal 95% CI, 0.526–0.955; raw *p* = 0.0239; Holm-adjusted *p* = 0.0478; Table 8). The binary analysis of MP grades 4–5 versus 1–3 provided a related assessment: adding PS to the HR–Ki67 model improved fit (likelihood ratio χ^2^ = 6.799, df = 1; Holm-adjusted *p* = 0.018). These findings suggest that PS carries response-related information not fully represented by HR and Ki67 in this sample. They make the combined-treatment setting a useful focus for further investigation. However, significance in this group and not in the chemotherapy-alone group does not establish a difference in PS–MP associations between treatment groups or show that PS predicts benefit from adding anti-HER2 therapy.

Bootstrap internal validation quantified optimism in the apparent discrimination of the binary response models. The apparent AUCs were 0.752, 0.687, and 0.781 for the PS, clinical, and combined models, respectively; the corresponding optimism-corrected estimates were 0.758, 0.649, and 0.742. The combined model retained a higher corrected point estimate than the clinical model but did not exceed PS alone. Adding HR and Ki67 jointly to the PS model also did not significantly improve fit (Holm-adjusted *p* = 0.236), which does not imply that these established markers lack clinical value. The likelihood ratio tests assess improvement in fit, whereas AUC describes discrimination. The results support further evaluation of the additional information from PS, without establishing superiority of the combined model or improved prediction in new patients.

One biological explanation worth examining concerns stromal effects on antibody transport and target accessibility. In human head and neck and pancreatic tumors, Lu et al. identified extracellular matrix- and fibroblast-rich neighborhoods associated with reduced delivery of labeled panitumumab [9]. More directly relevant to HER2-positive breast cancer, Liu et al. showed that CD16-positive fibroblasts can increase matrix stiffness and restrict trastuzumab delivery through a VAV2-dependent pathway [23]. Higher pericellular hyaluronan density has also been associated with reduced trastuzumab binding in human breast cancer tissue [24]. These studies suggest that the surrounding stroma may affect both antibody distribution and access to its target. A broader ultrasound region could capture tissue changes associated with these conditions, but our study did not measure drug delivery, target engagement, or tissue permeability.

The 1 mm interactions and the adjusted 7 mm association offer hypotheses about overlapping aspects of the peritumoral environment, rather than evidence of separate functional compartments. An overall PS association and a molecular interaction address different questions: a weak overall association can coexist with variation in that association across molecular profiles. However, the ROIs were nested cumulative regions, so the 7 mm ROI included the tissue sampled at narrower scales. The classifiers also differed in training/test membership and selected features. Differences among their outputs therefore cannot be attributed solely to distance from the tumor. Holm correction reduced the number of supported findings, but the exploratory analyses and data-dependent choices leave uncertainty about their reproducibility. Neither an optimal distance nor a specific biological function for any distance has been established.

Defining the tissue basis of these imaging associations could inform research into treatment resistance. In triple-negative breast cancer, Hofer et al. implicated an extracellular matrix-producing fibroblast subset in chemotherapy resistance involving SRC activation and G0S2 upregulation, using patient data and functional experiments [25]. Alongside the antibody-delivery studies, this suggests that stromal influences can involve both drug access and active resistance signaling. Studies combining pretreatment ultrasound with spatially matched pathology could test whether PS is associated with stromal composition, immune-cell organization, or vascular characteristics. Measurements of antibody distribution in patients receiving anti-HER2 therapy could then examine whether particular imaging phenotypes accompany barriers to effective drug exposure. Such studies would provide a mechanistic basis for interpreting PS and help identify resistance processes suitable for further investigation.

Several limitations remain. The response cohort was relatively small, and its formation depended on record availability and completion of treatment and surgery. Among the 728 patients with breast cancer, complete clinical records were unavailable for 332. Comparisons with the 396 patients with accessible records did not detect differences in age or tumor-size categories, but these limited comparisons cannot exclude selection bias. Molecular characteristics and subsequent outcomes could not be compared between the record-availability groups. Residual confounding by tumor size, tumor-infiltrating lymphocytes, BRCA1/2 alterations, and other factors may also have influenced the findings. HER2 status and treatment selection were closely linked, limiting separation of tumor biology from treatment effects.

Some response-cohort patients had contributed to training the malignancy classifiers. Although MP grade was not used to train those classifiers, this overlap prevents the full-cohort response analyses from serving as independent validation. In the scale-specific test subsets, all four OR point estimates were below 1, but their confidence intervals included 1 and none of the associations were significant after Holm correction. The estimates were compatible with the direction observed in the full cohort but did not independently confirm it. These subsets contained only 22–26 patients and differed in composition across scales, resulting in imprecise estimates. The 7 mm refit reduced direct overlap with SVM fitting and calibration by excluding all 132 response-cohort patients, but retained the original feature set, which had been selected with some of these patients present. The same response cases were used for the association analysis, and 7 mm had been selected for follow-up after the original multiscale analyses. Thus, the refit is a partial sensitivity analysis rather than independent validation of the complete pipeline. Strictly out-of-fold scores could not be regenerated from the platform, so uncertainty related to participation in upstream model training remains.

The bootstrap analysis addressed optimism from fitting the binary response models in the same 60 patients, conditional on the existing PS and the selected 7 mm scale. It did not repeat upstream classifier development or the process of selecting that scale for further analysis. Four of 1000 resamples produced separation in the clinical and combined models, and correction used the 996 resamples valid for all three models. The corrected AUCs remain point estimates without confidence intervals and do not establish between-model differences or clinical utility. Robustness to alternative ROI contours and mask definitions was also not evaluated. Independent evaluation of a fully specified pipeline is needed, with the scoring models, spatial scale, and response model fixed before evaluation and calibration assessed alongside discrimination.

With a larger, well-characterized response cohort, future work could develop models specifically trained to predict pathological response and evaluate them in independent, multicenter prospective cohorts. Integrating imaging with molecular characteristics and treatment information would allow a more complete assessment of added predictive value. Serial ultrasound could also determine whether early changes during therapy improve on pretreatment assessment, as suggested by recent multiparametric studies [18,19]. The present findings provide a focused rationale for these studies: an adjusted association at 7 mm in the chemotherapy plus anti-HER2 setting and molecular interactions at 1 mm. If reproduced and linked to interpretable tissue features, peritumoral PS could contribute to noninvasive assessment of treatment response and help guide further evaluation of inadequate response.

## 5. Conclusions

Higher 7 mm peritumoral ultrasound PS was associated with lower MP grade after multiplicity correction. At 1 mm, the PS–MP relationship varied with HR status and Ki67. In patients receiving chemotherapy plus anti-HER2 therapy, 7 mm PS remained associated with MP grade after adjustment and improved binary response model fit beyond HR and Ki67. The association persisted when the classifier was refitted using 1584 patients outside the response cohort. These exploratory findings support evaluating peritumoral PS as a complementary noninvasive marker of treatment response. Independent validation is needed in the future to establish its predictive performance and clinical value.

## Figures and Tables

**Table 1:** Clinical and pathological characteristics of 132 patients with breast cancer.

Characteristic	Category or Statistic	Value	Percentage (%)
Age (years)	Median	50	—
	Range	28–73	—
Age group (years)	<40	20	15.15
	40–49	42	31.82
	50–59	54	40.91
	≥60	16	12.12
Tumor size (mm)	≤10	3	2.27
	>10 to ≤20	67	50.76
	>20 to ≤50	56	42.42
	>50	6	4.55
Neoadjuvant treatment	Chemotherapy alone	70	53.03
	Chemotherapy plus anti-HER2 therapy	62	46.97
HER2 IHC score	0	25	18.94
	1+	26	19.70
	2+	29	21.97
	3+	52	39.39
HR	Positive	77	58.33
	Negative	55	41.67
Ki67 (%, *n* = 129)	Median (Q1–Q3)	40 (20–70)	—
	Mean ± SD	43.2 ± 26.3	—
	Range	1–90	—
MP grade	1	16	12.12
	2	24	18.18
	3	24	18.18
	4	14	10.61
	5	54	40.91

Note: Categorical data are reported as counts and percentages, with 132 as the denominator. Percentages may not sum to 100% because of rounding. Age is summarized as median and range. Ki67 data were available for 129 patients and missing for three; available values are summarized as median (Q1–Q3), mean ± SD, and range, with available observations. MP grade was assessed in the surgical specimen after neoadjuvant therapy. HER2, human epidermal growth factor receptor 2; IHC, immunohistochemistry; HR, hormone receptor; MP, Miller–Payne; Q1, first quartile; Q3, third quartile; SD, standard deviation.

**Table 2:** Distribution of PS at four peritumoral scales in 132 patients.

Peritumoral Distance (mm)	Mean ± SD	Median	Q1–Q3
1	0.668 ± 0.195	0.729	0.500–0.826
3	0.687 ± 0.165	0.731	0.605–0.812
5	0.704 ± 0.175	0.774	0.611–0.814
7	0.690 ± 0.180	0.751	0.569–0.811

Note: All four scales include the same 132 patients. Peritumoral distance denotes outward expansion from the tumor boundary; each ROI is a cumulative peritumoral region excluding the tumor interior. Higher PS indicates a stronger model-assigned tendency toward lesion malignancy. PS, prediction score; SD, standard deviation; Q1, first quartile; Q3, third quartile; ROI, region of interest.

**Table 3:** Univariable ordinal logistic associations between peritumoral PS and MP grade.

Peritumoral Distance (mm)	OR per 0.1-Unit Increase in PS	95% CI	Raw *p* Value	Holm-Adjusted *p* Value
1	0.87	0.74–1.02	0.079	0.079
3	0.80	0.66–0.97	0.022	0.066
5	0.82	0.68–0.99	0.037	0.074
7	0.79	0.66–0.95	0.012	**0.048**

Note: Separate univariable ordinal logistic models were fitted in 132 patients. ORs represent the odds of a higher MP grade per 0.1-unit increase in PS; OR < 1 indicates lower odds as PS increases. Holm correction was applied across the four overall PS–MP association tests. Raw and adjusted *p* values are shown; bold indicates Holm-adjusted *p* < 0.05. PS, prediction score; MP, Miller–Payne grade; OR, odds ratio; CI, confidence interval.

**Table 4:** Interaction between peritumoral PS and HR status in relation to MP grade.

Peritumoral Distance (mm)	Interaction β	Interaction OR	95% CI	Raw *p* Value	Holm-Adjusted *p* Value
1	0.588	1.80	1.20–2.71	0.005	**0.049**
3	0.310	1.36	0.91–2.04	0.130	0.618
5	0.323	1.38	0.94–2.04	0.103	0.618
7	0.239	1.27	0.86–1.87	0.228	0.684

Note: At each scale, an ordinal logistic model included PS, HR status, and PS × HR, with MP grade as the outcome. PS was scaled per 0.1-unit increase, and HR-negative status was the reference. β, OR, 95% CI, and *p* values refer to the interaction term. The interaction OR is the ratio of the PS effect OR in HR-positive patients to that in HR-negative patients. Holm correction was applied across 12 interaction tests involving HR, continuous Ki67, and dichotomized Ki67 at four scales (Table 4, Table 5 and Table S5). Bold indicates Holm-adjusted *p* < 0.05. PS, prediction score; HR, hormone receptor; MP, Miller–Payne grade; OR, odds ratio; CI, confidence interval.

**Table 5:** Interaction between peritumoral PS and continuous Ki67 in relation to MP grade.

Peritumoral Distance (mm)	Interaction β	Interaction OR	95% CI	Raw *p* Value	Holm-Adjusted *p* Value
1	−0.106	0.90	0.84–0.97	0.003	**0.036**
3	−0.098	0.91	0.84–0.98	0.019	0.171
5	−0.082	0.92	0.85–1.00	0.060	0.420
7	−0.035	0.97	0.89–1.05	0.395	0.790

Note: Separate ordinal logistic models were fitted in 129 complete cases. PS was scaled per 0.1-unit increase and Ki67 per 10-percentage-point increase. β, OR, and 95% CI refer to PS × Ki67. The interaction OR is the multiplicative change in the PS effect OR for each 10-percentage-point increase in Ki67. Holm correction was applied across 12 interaction tests involving HR, continuous Ki67, and dichotomized Ki67 at four scales (Table 4, Table 5 and Table S5). Bold indicates Holm-adjusted *p* < 0.05. PS, prediction score; MP, Miller–Payne grade; OR, odds ratio; CI, confidence interval.

**Table 6:** Treatment-stratified multivariable ordinal logistic regression of peritumoral PS in relation to MP grade.

Treatment Regimen	Model	Variable	Adjusted OR	95% CI	Raw *p* Value	Holm-Adjusted *p* Value
Chemotherapy alone (*n* = 69)	Model 1	1 mm PS	0.90	0.73–1.12	0.340	1.000
		HR positive (vs. negative)	1.18	0.39–3.62	0.769	—
		Ki67	1.16	0.97–1.38	0.095	—
	Model 2	3 mm PS	0.91	0.70–1.19	0.486	1.000
		HR positive (vs. negative)	1.08	0.35–3.29	0.895	—
		Ki67	1.15	0.97–1.36	0.115	—
	Model 3	5 mm PS	0.96	0.73–1.26	0.746	1.000
		HR positive (vs. negative)	1.11	0.36–3.37	0.859	—
		Ki67	1.14	0.96–1.36	0.131	—
	Model 4	7 mm PS	0.99	0.76–1.29	0.953	1.000
		HR positive (vs. negative)	1.10	0.36–3.37	0.861	—
		Ki67	1.14	0.96–1.34	0.140	—
Chemotherapy plus anti-HER2 therapy (*n* = 60)	Model 1	1 mm PS	0.84	0.64–1.10	0.201	1.000
		HR positive (vs. negative)	0.35	0.12–1.00	0.051	—
		Ki67	1.20	0.94–1.53	0.150	—
	Model 2	3 mm PS	0.76	0.52–1.11	0.160	0.960
		HR positive (vs. negative)	0.41	0.14–1.22	0.109	—
		Ki67	1.16	0.90–1.49	0.250	—
	Model 3	5 mm PS	0.75	0.55–1.03	0.080	0.560
		HR positive (vs. negative)	0.40	0.13–1.16	0.091	—
		Ki67	1.19	0.93–1.52	0.156	—
	Model 4	7 mm PS	0.60	0.42–0.85	0.005	**0.03** **7**
		HR positive (vs. negative)	0.43	0.14–1.27	0.128	—
		Ki67	1.20	0.93–1.54	0.163	—

Note: Models 1–4 include PS at 1, 3, 5, and 7 mm, respectively, together with HR and continuous Ki67. Complete-case sample sizes are 69 for chemotherapy alone and 60 for chemotherapy plus anti-HER2 therapy. PS ORs are per 0.1-unit increase, Ki67 ORs per 10-percentage-point increase, and HR-negative status is the reference. OR > 1 indicates higher odds and OR < 1 lower odds of a higher MP grade. Holm correction was applied across the eight PS tests spanning four scales and two treatment groups. HR and Ki67 are adjustment terms; their raw *p* values are shown, and “—” indicates that they were not included in this correction family. Bold indicates Holm-adjusted *p* < 0.05. PS, prediction score; MP, Miller–Payne grade; HR, hormone receptor; HER2, human epidermal growth factor receptor 2; OR, odds ratio; CI, confidence interval.

**Table 7:** Likelihood ratio comparisons of nested models for high pathological response.

Comparison	Added Predictor(s)	LR χ^2^	df	Raw *p* Value	Holm-Adjusted *p* Value
Clinical → Combined	7 mm PS	6.799	1	0.00912	**0.01824**
PS7 → Combined	HR and Ki67	2.891	2	0.23568	0.23568

Note: All models were fitted to the same 60 complete cases receiving chemotherapy plus anti-HER2 therapy. High response was MP grades 4–5 (*n* = 43) and low response MP grades 1–3 (*n* = 17). The clinical model included HR and continuous Ki67; the PS model included 7 mm PS; the combined model included all three predictors. The likelihood ratio statistic is twice the difference in maximized log-likelihoods between nested models. Holm correction was applied across the two comparisons; bold indicates Holm-adjusted *p* < 0.05. These tests assess model fit, not differences between apparent or optimism-corrected AUCs. LR, likelihood ratio; df, degrees of freedom; PS, prediction score; HR, hormone receptor; MP, Miller–Payne; AUC, area under the receiver operating characteristic curve.

**Table 8:** Treatment-stratified multivariable ordinal logistic regression of refitted 7 mm PS in relation to MP grade.

Treatment Regimen	Variable	Adjusted OR	95% CI	Raw *p* Value	Holm-Adjusted *p* Value
Chemotherapy alone (*n* = 69)	Refitted 7 mm PS	0.938	0.743–1.184	0.5896	0.5896
	HR positive	1.073	0.350–3.290	0.9022	—
	Ki67	1.141	0.963–1.352	0.1262	—
Chemotherapy plus anti-HER2 therapy (*n* = 60)	Refitted 7 mm PS	0.709	0.526–0.955	0.0239	**0.0** **478**
	HR positive	0.354	0.123–1.022	0.0549	—
	Ki67	1.175	0.915–1.508	0.2071	—

Note: The models used the same complete-case samples as the original treatment-stratified 7 mm analysis: 69 patients receiving chemotherapy alone and 60 receiving chemotherapy plus anti-HER2 therapy. Each model included refitted 7 mm PS, HR status, and continuous Ki67. PS ORs are per 0.1-unit increase; Ki67 ORs are per 10-percentage-point increase; HR-negative status is the reference. Reported 95% CIs are nominal. Holm correction was applied to the two refitted PS coefficient tests, one in each treatment group. HR and Ki67 are adjustment terms; their raw *p* values are shown, and “—“ indicates that they were not included in the two-test correction family. Bold indicates Holm-adjusted *p* < 0.05. PS, prediction score; MP, Miller–Payne grade; HR, hormone receptor; OR, odds ratio; CI, confidence interval.

## Data Availability

The data presented in this study are available on request from the corresponding author.

## Supplementary Materials

The following supporting information can be downloaded at: https://www.mdpi.com/article/10.3390/diagnostics16213580/s1. Table S1: Baseline characteristics by clinical-record availability, with the response cohort shown for descriptive context; Table S2: Univariable ordinal logistic associations between PS and MP grade in the scale-specific test subsets; Table S3: Spearman correlations between peritumoral PS and MP grade stratified by HER2 IHC score; Table S4: Spearman correlations between peritumoral PS and MP grade stratified by HR status; Table S5: Interaction between peritumoral PS and dichotomized Ki67 (<30% vs. ≥30%) in relation to MP grade; Table S6: Ordinal logistic associations between peritumoral PS and MP grade stratified by Ki67 group; Table S7: Apparent and optimism-corrected discrimination of high pathological response in the chemotherapy plus anti-HER2 group.

## Abbreviations

The following abbreviations are used in this manuscript:

AI	Artificial intelligence
AUC	Area under the receiver operating characteristic curve
BRCA1/2	Breast cancer susceptibility genes 1 and 2
CDK4/6	Cyclin-dependent kinases 4 and 6
CI	Confidence interval
ER	Estrogen receptor
FISH	Fluorescence in situ hybridization
GenAI	Generative artificial intelligence
HER2	Human epidermal growth factor receptor 2
HR	Hormone receptor
IHC	Immunohistochemistry
LASSO	Least absolute shrinkage and selection operator
MP	Miller–Payne
NAT	Neoadjuvant therapy
OR	Odds ratio
PR	Progesterone receptor
PS	Prediction score
Q1/Q3	First/third quartile
ROC	Receiver operating characteristic
ROI	Region of interest
SD	Standard deviation
SVM	Support vector machine
TILs	Tumor-infiltrating lymphocytes
VEGFR	Vascular endothelial growth factor receptor
