# Supplementary material for: Multiscale Peritumoral Ultrasound Machine Learning Scores and Pathological Response to Neoadjuvant Therapy in Breast Cancer
Source: Diagnostics (Basel). 2026 Oct 29;16(21):3580. doi: 10.3390/diagnostics16213580 (PMC13761895; doi:10.3390/diagnostics16213580)
Supplement: Supplementary file 1 [file diagnostics-16-03580-s001.zip › diagnostics-4628671-supplementary.pdf]

Supplementary Materials

Table S1. Baseline characteristics by clinical-record availability, with the response cohort shown for descriptive context.

Characteristic	Category or statistic	Response cohort (n = 132)	Accessible records (n = 396)	Inaccessible records (n = 332)	P value*
Age (years)	Median	50	52	53	0.110
	Range	28–73	28–79	26–75	
Age group (years)	<40	20	55	48	0.457
	40–49	42	103	72	
	50–59	54	182	159	
	≥60	16	56	53	
Tumor size (mm)	≤10	3	20	13	0.177
	>10 to ≤20	67	203	165	
	>20 to ≤50	56	158	132	
	>50	6	15	22	
HER2 IHC score	0	25	53	—	—
	1+	26	78	—	—
	2+	29	90	—	—
	3+	52	175	—	—
HR	Positive	77	267	—	—
	Negative	55	129	—	—
Ki67 (%)	Median (Q1–Q3)	40 (20–70)	42 (20–73)	—	—
	Mean ± SD	43.2 ± 26.3	44.0 ± 25.8	—	—
	Range	1–90	1–95	—	—

Note: The comparison concerns record availability among 728 patients with breast cancer: 396 had accessible complete clinical history records and 332 did not. The response cohort (n = 132) is a subset of the 396-patient group and is shown only for descriptive context. Counts are reported for categorical variables. P values compare the 396- and 332-patient groups only; ordered age and tumor-size categories were evaluated using Mantel–Haenszel tests for ordinal association. No multiplicity correction was applied to these descriptive baseline comparisons. HER2, HR, and Ki67 data were unavailable in the 332-patient group, so no between-group comparisons were performed for these variables. "—" denotes unavailable data or a comparison not performed. Ki67 summaries were based on available observations: 129 of 132 patients in the response cohort and 388 of 396 patients in the accessible-record group. HER2, human epidermal growth factor receptor 2; IHC, immunohistochemistry; HR, hormone receptor; Q1 and Q3, first and third quartiles; SD, standard deviation. *, P values compare patients with accessible clinical records (n = 396) with those whose records were inaccessible (n = 332). The response cohort (n = 132) is a subset of the accessible-record group and is shown for descriptive purposes; it was not treated as a separate group in these comparisons.

Table S2. Univariable ordinal logistic associations between PS and MP grade in the scale-specific test subsets.

Peritumoral distance (mm)	n	β	OR per 0.1-unit increase in PS	95% CI	Raw P value	Holm-adjusted P value
1	26	-0.059	0.942	0.681–1.304	0.721	0.888
3	22	-0.454	0.635	0.398–1.012	0.056	0.224
5	23	-0.354	0.702	0.466–1.058	0.091	0.273
7	25	-0.163	0.850	0.560–1.289	0.444	0.888

Note: Each analysis included only response-cohort patients assigned to the test set of the corresponding malignancy classifier. Patient membership differed across scales; these are four scale-specific subsets, not one common test cohort. Separate univariable ordinal logistic models used MP grade (1–5) as the outcome. β and OR refer to a 0.1-unit increase in PS, with OR <1 indicating lower odds of a higher MP grade. Holm correction was applied across these four tests. No adjusted P value was <0.05. The 95% CIs are nominal and were not adjusted for multiplicity. PS, prediction score; MP, Miller–Payne; OR, odds ratio; CI, confidence interval.

Table S3. Spearman correlations between peritumoral PS and MP grade stratified by HER2 IHC score.

HER2 IHC score	n	Peritumoral distance (mm)	Spearman ρ	Raw P value	Holm-adjusted P value
0	25	1	-0.055	0.795	1.000
		3	-0.085	0.686	1.000
		5	0.076	0.718	1.000
		7	0.136	0.518	1.000
1+	26	1	-0.090	0.662	1.000
		3	0.031	0.881	1.000
		5	0.184	0.367	1.000
		7	0.163	0.427	1.000
2+	29	1	0.023	0.905	1.000
		3	-0.135	0.486	1.000
		5	0.101	0.604	1.000
		7	-0.174	0.368	1.000
3+	52	1	-0.214	0.128	1.000
		3	-0.197	0.162	1.000
		5	-0.303	0.029	0.725
		7	-0.375	0.006	0.174

Note: The analysis included 132 patients, with 25, 26, 29, and 52 in the HER2 IHC 0, 1+, 2+, and 3+ strata, respectively. Within each stratum, Spearman correlations were calculated between MP grade and PS at 1, 3, 5, and 7 mm. Negative ρ indicates an inverse association, and the stratum sample size applies to all four scales. Holm correction was applied across 32 molecular subgroup tests: 16 HER2-stratified correlations, eight HR-stratified correlations, and eight Ki67-stratified ordinal regression associations (Tables S3, S4, and S6). No correlation in this table had an adjusted $P < 0.05$. HER2, human epidermal growth factor receptor 2; IHC, immunohistochemistry; PS, prediction score; MP, Miller–Payne grade.

Table S4. Spearman correlations between peritumoral PS and MP grade stratified by HR status.

HR status	n	Peritumoral distance (mm)	Spearman ρ	Raw P value	Holm-adjusted P value
Negative	55	1	-0.398	0.003	0.090
		3	-0.333	0.013	0.338
		5	-0.279	0.039	0.936
		7	-0.230	0.092	1.000
Positive	77	1	-0.011	0.926	1.000
		3	-0.149	0.195	1.000
		5	-0.013	0.913	1.000
		7	-0.118	0.307	1.000

Note: The analysis included 132 patients: 55 HR-negative and 77 HR-positive. HR positivity required positivity for either ER or PR; tumors negative for both were HR-negative. Correlations were calculated separately at each scale within each HR group. Negative ρ indicates an inverse association. The group sample size applies to all four scales. Holm correction was applied across the 32 molecular subgroup tests in Tables S3, S4, and S6. No correlation in this table had an adjusted $P < 0.05$. Between-group differences in PS effects were assessed separately by the interaction models in Table 4. HR, hormone receptor; ER, estrogen receptor; PR, progesterone receptor; PS, prediction score; MP, Miller–Payne grade.

Table S5. Interaction between peritumoral PS and dichotomized Ki67 (<30% vs. ≥30%) in relation to MP grade.

Peritumoral distance (mm)	Interaction β	Interaction OR	95% CI	Raw P value	Holm-adjusted P value
1	-0.493	0.611	0.438–0.851	0.004	**0.044**
3	-0.482	0.617	0.411–0.928	0.020	0.171
5	-0.308	0.735	0.499–1.081	0.118	0.618
7	-0.163	0.850	0.580–1.244	0.402	0.790

Note: The analysis included 129 complete cases: Ki67 <30% (reference group, n = 46) and Ki67 ≥30% (n = 83). Each ordinal logistic model included PS, Ki67 group, and their interaction, with MP grade as the outcome. PS was scaled per 0.1-unit increase. β, OR, CI, and P values refer to the interaction term. The interaction OR is the ratio of the PS effect OR in the Ki67 ≥30% group to that in the Ki67 <30% group. Holm correction was applied across the 12 interaction tests in Tables 4, 5, and S5. Bold indicates Holm adjusted P < 0.05. PS, prediction score; MP, Miller–Payne grade; OR, odds ratio; CI, confidence interval.

Table S6. Ordinal logistic associations between peritumoral PS and MP grade stratified by Ki67 group.

Ki67 group	n	Peritumoral distance (mm)	OR per 0.1-unit increase in PS	95% CI	P value	Holm-adjusted P value
<30%	46	1	1.08	0.87-1.35	0.493	1.000
		3	1.01	0.70–1.45	0.957	1.000
		5	0.95	0.71–1.28	0.734	1.000
		7	0.84	0.62–1.14	0.257	1.000
≥30%	83	1	0.67	0.53–0.85	0.001	**0.032**
		3	0.63	0.47–0.84	0.002	0.055
		5	0.71	0.55–0.92	0.009	0.252
		7	0.73	0.57–0.93	0.012	0.324

Note: The analysis included 129 complete cases: 46 with Ki67 <30% and 83 with Ki67 ≥30%. Within each group, separate ordinal logistic models assessed PS at each scale in relation to MP grade. ORs represent the odds of a higher MP grade per 0.1-unit increase in PS; OR <1 indicates lower odds with increasing PS. Sample sizes apply to all four scales within each group. Holm correction was applied across the 32 molecular subgroup tests in Tables S3, S4, and S6. Between-group differences are assessed by the interaction terms in Table S5. Bold indicates Holm adjusted $P < 0.05$. PS, prediction score; MP, Miller–Payne grade; OR, odds ratio.

Table S7. Apparent and optimism-corrected discrimination of high pathological response in the chemotherapy plus anti-HER2 group.

Model	Apparent AUC	95% CI for apparent AUC	Optimism-corrected AUC
7 mm PS model	0.752	0.620–0.885	0.758
Clinical model	0.687	0.521–0.854	0.649
Combined model	0.781	0.650–0.912	0.742

Note: All models used the same 60 complete cases receiving chemotherapy plus anti-HER2 therapy, comprising 43 patients with MP grades 4–5 and 17 with MP grades 1–3. The PS model included 7 mm PS alone, the clinical model HR and continuous Ki67, and the combined model all three predictors. Apparent AUCs were calculated from fitted probabilities in the development sample; the nominal 95% CIs were calculated using the DeLong method and apply only to the apparent AUCs. Internal validation used 1000 ordinary patient-level bootstrap resamples. Four resamples with separation in the clinical and combined models were excluded from all three optimism summaries, leaving 996 common valid resamples. Optimism-corrected AUCs were calculated by subtracting mean optimism from apparent AUCs. The slightly higher corrected AUC for the PS model reflects a negative mean optimism estimate. Corrected AUCs are point estimates without CIs; validation was conditional on the existing PS and the selected 7 mm scale. AUC, area under the receiver operating characteristic curve; CI, confidence interval; PS, prediction score; HR, hormone receptor; MP, Miller–Payne; HER2, human epidermal growth factor receptor 2.
